# Cardiovascular Health Among High School Students — Youth Risk Behavior Survey, United States, 2025

**DOI:** 10.15585/mmwr.su7501a3

**Published:** 2026-10-15

**Authors:** Izraelle I. McKinnon, Ryan Saelee, Kathleen H. Krause, Domonique Hargrave, Caitlin Merlo, Shannon L. Michael, Philip E. Rosenbaum, Ahlia Sekkarie, Christopher J. Kissler, Jonetta J. Mpofu

**Affiliations:** ^1^Division of Adolescent and School Health, National Center for Chronic Disease Prevention and Health Promotion, CDC, Atlanta, Georgia; ^2^U.S. Public Health Service, Rockville, Maryland; ^3^Division for Heart Disease and Stroke Prevention, National Center for Chronic Disease Prevention and Health Promotion, CDC, Atlanta, Georgia

## Abstract

Monitoring cardiovascular health (CVH) among adolescents can identify opportunities for school and community programs to promote healthy behaviors and reduce cardiovascular disease risk. In this report, data from the nationally representative 2025 Youth Risk Behavior Survey (YRBS) of U.S. high school students were used to calculate scores for five metrics of CVH aligned with the American Heart Association’s Life’s Essential 8 framework: diet, physical activity, nicotine exposure, sleep health, and body mass index (BMI). Each of the five metrics assessed in this analysis was scored from 0 to 100, with higher scores indicating more favorable levels of the corresponding metric. The five metric scores were averaged to produce an overall CVH score, with higher scores indicating a more favorable overall CVH score. Weighted mean scores and the prevalence of high (80–100), moderate (50–79), and low (0–49) scores were estimated overall and by sex, grade, and race and ethnicity.

Among 5,192 students with complete data, the mean overall CVH score was 65.3; 20.7% had high, 61.3% had moderate, and 18.0% had low overall CVH scores. For the CVH metrics, mean scores were highest for BMI (85.4, reflecting lower BMI) and nicotine exposure (80.5, reflecting lower exposure), and lowest for diet (39.3), sleep health (59.1), and physical activity (62.0). The overall CVH score was lower among female (64.2) than male (66.2) students. The overall CVH score was higher for grade 9 students (68.3) than for grade 10 (65.9), 11 (63.7), and 12 (62.7) students. Compared with most other racial and ethnic groups, the overall CVH score was higher among Asian students (70.2) and lower among Black or African American students (60.2). Similar demographic patterns were observed for the prevalence of high overall CVH scores.

In 2025, approximately four in five U.S. high school students had a low or moderate overall CVH score, underscoring the importance of promoting healthy adolescent behaviors, such as healthy eating, sleep, and physical activity, to support healthier trajectories into adulthood. These findings highlight opportunities to monitor and improve CVH-related behaviors and indicators among adolescents, including within school and community settings.

## Introduction

Cardiovascular diseases (CVDs), which include conditions such as coronary heart disease, heart failure, stroke, and hypertension (*1*), remain leading causes of death in the United States (*2*), and many risk factors for CVD originate during childhood and adolescence (*3*). The American Heart Association (AHA) has emphasized preventing CVD by promoting modifiable lifestyle choices that contribute to cardiovascular health (CVH) (*3*). AHA’s Life’s Essential 8 (LE8) defines CVH using eight metrics, including four health behaviors (diet, physical activity, nicotine exposure, and sleep health) and four health factors (weight status assessed by body mass index [BMI], blood lipid level, blood pressure, and blood glucose level), which are key modifiable determinants of risk for CVD (*4*). National data indicate that optimal CVH is uncommon and has declined among U.S. adults (*5*), and the prevalence of obesity, prediabetes, and other CVD risk factors has increased among adolescents (*1*,*3*).

Adolescence is a critical period for the development of health behaviors that can persist into adulthood and influence long-term cardiovascular outcomes (*6*,*7*). Diet, physical activity, sleep, nicotine exposure, and BMI are each independently associated with CVD and are also interrelated, making their combined assessment important for understanding overall CVH (*4*,*6*). Differences in CVH-related behaviors and health factors have been observed by sex, age, and race and ethnicity. Previous reports have indicated that female students report less physical activity and are less likely to report sufficient sleep duration than male students (https://www.cdc.gov/yrbs/dstr/pdf/DSTR-Dietary-PhysicalActivity-SleepBehavior2013-2023-508.pdf), and that multiple CVH-related behaviors decline as adolescents get older (*1*,*7*,*8*). Patterns in these behaviors and health factors also vary among racial and ethnic groups (*1*,*4*,*7*,*8*). These differences highlight the importance of examining CVH by sex, grade, and race and ethnicity. National public health priorities, including Healthy People 2030 (https://odphp.health.gov/healthypeople), emphasize improving nutrition, physical activity, sleep, and tobacco use outcomes among adolescents, aligning with the core metrics of CVH.

The Youth Risk Behavior Surveillance System (YRBSS), the largest school-based surveillance system in the United States, provides data on these behaviors among high school students and includes nationally representative data as well as data from states, territories, tribal governments, and local school districts (*9*). This report analyzed data from the 2025 national Youth Risk Behavior Survey (YRBS), a component of YRBSS, to estimate five CVH metrics (diet, physical activity, nicotine exposure, sleep health, and BMI) and an overall CVH score among U.S. high school students. Differences in CVH metric scores and the overall CVH score were examined by sex, grade, and race and ethnicity. These findings are intended for public health practitioners, school and education professionals, researchers, and policymakers to guide efforts for monitoring CVH among adolescents and strengthen school and community policies and practices that support healthy behaviors.

## Methods

### Data Source

This report includes data from the 2025 YRBS (N = 10,710), a cross-sectional, school-based survey conducted biennially since 1991. Each survey year, CDC collects data from a nationally representative sample of students from public and private schools, including parochial schools, in grades 9–12 in the 50 U.S. states and the District of Columbia. Additional information about YRBS sampling, data collection, response rates, and processing is available in the overview report of this supplement (*9*). The prevalence estimates for all YRBS variables among U.S. high school students overall and stratified by demographic characteristics are available at https://youthonline.cdc.gov. The full YRBS questionnaire, datasets, and documentation are available at https://www.cdc.gov/yrbs/index.html. This study was approved by institutional review boards at CDC and ICF, the survey contractor, and was conducted consistent with applicable federal law and CDC policy.*

### Measures

CVH was assessed using five metrics aligned with LE8: diet, physical activity, nicotine exposure, sleep health, and BMI. Because YRBS does not include blood collection or a physical examination, blood lipid levels, blood glucose levels, and blood pressure were not assessed.

Each CVH metric was scored on a scale from 0 to 100, with higher scores indicating more favorable health behaviors or factors. Physical activity and BMI were scored using algorithms consistent with AHA guidance for children and persons aged 2–19 years (*4*). BMI was calculated from self-reported height and weight and converted to age- and sex-specific percentiles using CDC growth charts (https://www.cdc.gov/growthcharts/cdc_charts.htm). Sleep health was scored using the recommended sleep duration of 8–10 hours per night for adolescents. However, the highest YRBS response category is “10 or more hours”; therefore, students reporting >10 hours of sleep, which is above the recommended range, could not be distinguished from those reporting exactly 10 hours, which is within the recommended range. Overall, 1.7% of students reported ≥10 hours of sleep on an average school night (unweighted n = 164).

Diet and nicotine exposure metrics were constructed using available YRBS measures to approximate LE8 scoring algorithms; however, certain LE8 components were not available in YRBS for these metrics. For example, the diet metric was based on reported consumption of fruits, vegetables, soda and sports drinks and does not include other components of diet (e.g., whole grains). The nicotine exposure metric did not include secondhand smoke exposure in the home. Additional details on the construction and scoring of each CVH metric are provided (Table 1).

**TABLE 1:** Questions, response options, and analytic coding for Youth Risk Behavior Survey measures used to construct cardiovascular health metrics aligned with American Heart Association's Life's Essential 8 — Youth Risk Behavior Survey, United States, 2025

CVH metric	Question	Response option	Analytic coding
Diet*			
Fruit	During the past 7 days, how many times did you eat fruit? (Do not count fruit juice.)	I did not eat or drink it during the past 7 days, ate or drank it 1–3 times during the past 7 days, ate or drank it 4–6 times during the past 7 days, ate or drank it 1 time per day, ate or drank it 2 times per day, ate or drank it 3 times per day, or ate or drank it ≥4 times per day	• 10 points for intake ≥2 times per day • 5 points for intake 1 time per day • 0 points for <1 time per day
Vegetables	• During the past 7 days, how many times did you eat green salad? • During the past 7 days, how many times did you eat potatoes? (Do not count French fries, fried potatoes, or potato chips.) • During the past 7 days, how many times did you eat carrots? • During the past 7 days, how many times did you eat other vegetables? (Do not count green salad, potatoes, or carrots.)		• 10 points for intake ≥3 times per day • 5 points for intake ≥1 to <3 times per day • 0 points for <1 time per day
Sugar-sweetened beverages	• During the past 7 days, how many times did you drink a can, bottle, or glass of soda or pop, such as Coke, Pepsi, or Sprite? (Do not count diet soda or diet pop.) • During the past 7 days, how many times did you drink a can, bottle, or glass of sports drink such as Gatorade or Powerade? (Do not count low-calorie sports drinks such as Propel or G2.)		• 10 points for intake 0 times per week • 5 points for intake >0 times per week to <1 time per day • 0 points for ≥1 time per day
**Physical activity^†^**	During the past 7 days, on how many days were you physically active for a total of at least 60 minutes per day? (Add up all the time you spent in any kind of physical activity that increased your heart rate and made you breathe hard some of the time.)	0 days, 1 day, 2 days, 3 days, 4 days, 5 days, 6 days, or 7 days	• 100 points for 7 days • 90 points for 6 days • 80 points for 5 days • 60 points for 4 days • 40 points for 2 or 3 days • 20 points for 1 day • 0 points for 0 days
**Nicotine exposure^§^**	• Have you ever smoked a cigarette, even one or two puffs? • During the past 30 days, on how many days did you smoke cigarettes? • Have you ever used an electronic vapor product? • During the past 30 days, on how many days did you use an electronic vapor product? • During the past 30 days, on how many days did you smoke cigars, cigarillos, or little cigars, such as Swisher Sweets, Middleton's (including Black and Mild), or Backwoods?	**For ever measures** Yes or No **For past 30 days measures** 0 days, 1 or 2 days, 3–5 days, 6–9 days, 10–19 days, 20–29 days, or all 30 days	• 100 points for never tried • 50 points for tried any nicotine product, but >30 days ago • 25 points for current use of an electronic vapor product • 0 points for current use of combustible products
**Sleep health^¶^**	On an average school night, how many hours of sleep do you get?	≤4 hrs, 5 hrs, 6 hrs, 7 hrs, 8 hrs, 9 hrs, or ≥10 hrs	• 100 points for 8, 9, or ≥10 hrs • 70 points for 7 hrs • 40 points for 6 hrs • 20 points for 5 hrs • 0 points for ≤4 hrs
BMI**	• How tall are you without your shoes on? • How much do you weigh without shoes on? • How old are you? • What is your sex?	**Height and weight are free-text entry** **Age** ≤12 yrs, 13 yrs, 14 yrs, 15 yrs, 16 yrs, 17 yrs, or ≥18 yrs **Sex** Female or male	• 100 points for <85th percentile • 70 points for 85th to <95th percentile • 30 points for 95th percentile to <120% of the 95th percentile • 15 points for 120% of the 95th percentile to <140% of the 95th percentile • 0 points for ≥140% of the 95th percentile

**Abbreviations:** BMI = body mass index; CVH = cardiovascular health; LE8 = American Heart Association’s Life's Essential 8; YRBS = Youth Risk Behavior Survey.

* YRBS assesses dietary behaviors using self-reported frequency of fruit, vegetable, and sugar-sweetened beverage (soda and sports drink) consumption. These YRBS measures overlap with components included in LE8 dietary scoring approaches (e.g., Dietary Approaches to Stop Hypertension, Healthy Eating Index, and Mediterranean Eating Patterns for Americans), although several components of these comprehensive assessments are not collected in YRBS. Consistent with these approaches, dietary components were equally weighted to construct a composite score. An allocation of 10 points indicates more favorable practice and an allocation of 0 points indicates less favorable practice.

All fruit, vegetable, soda, and sports drink items used the same seven response options shown. For scoring, responses were converted to estimated intake frequency in times per day. “I did not eat/drink [item] during the past 7 days” was assigned 0 times per day; “1 to 3 times during the past 7 days” was assigned 2/7 times per day; and “4 to 6 times during the past 7 days” was assigned 5/7 times per day. Responses of 1, 2, 3, or ≥4 times per day were assigned their corresponding daily frequencies. Thus, the scoring category “<1 time/day” is a derived category and includes the weekly-frequency response options.

For fruit intake, the single-item measure of fruit consumption was categorized directly using the response options. For vegetables, the estimated times-per-day values from the four vegetable items were summed to create a composite vegetable intake estimate. For sugar-sweetened beverages, the estimated times-per-day values from the soda and sports drink items were summed to create a composite sugar-sweetened beverage intake estimate. The fruit, vegetable, and sugar-sweetened beverage intake estimates were then categorized using the thresholds shown and assigned 0, 5, or 10 points. Points for the three dietary components were averaged and multiplied by 10 to create a composite diet score of 0–100.

The Dietary Guidelines for Americans recommend approximately two servings of fruit and three servings of vegetables per day (https://www.dietaryguidelines.gov/). Accordingly, ideal intake was defined as ≥2 times per day for fruit and ≥3 times per day for vegetables (10 points each). Intake <1 time per day was considered least ideal (0 points), and intermediate intake levels were assigned 5 points. Previous research comparing frequency-based survey measures with 24-hour dietary recall data indicates that times per day does not directly correspond to number of servings per day and might overestimate actual consumption (**Source:** Eaton DK, Olsen EO, Brener ND, et al. A comparison of fruit and vegetable intake estimates from three survey question sets to estimates from 24-hour dietary recall interviews J Acad Nutr Diet 2013;113:1165–74). Because frequency does not directly correspond to quantity consumed, and recommendations vary by sex, body size, and physical activity level, this approach might overestimate adherence to dietary guidelines. For sugar-sweetened beverages, no consumption was considered ideal (10 points), whereas ≥1 time per day was least ideal (0 points); intermediate intake levels were assigned 5 points.

^†^ YRBS's time-based measure of days of at least 60 minutes of aerobic physical activity aligns with LE8's recommended scoring for moderate to vigorous physical activity in minutes per week. Although YRBS also includes data for muscle strengthening activity aligned with overall physical activity recommendations for adolescents, this activity is not explicitly part of LE8's scoring recommendations for children and adolescents as related to CVH. An allocation of 100 points indicates more favorable practice and an allocation of 0 points indicates less favorable practice.

^§^ Consistent with the LE8 nicotine exposure scoring approach, current use of combustible nicotine products was assigned a lower score than current use of electronic vapor products. Accordingly, students reporting current combustible product use, including cigarettes or cigars, were assigned 0 points, whereas students reporting current electronic vapor product use but no current combustible product use were assigned 25 points. Students who had tried cigarettes or electronic vapor products but had not used them in the past 30 days were assigned 50 points, and students who had never tried either product were assigned 100 points. Although YRBS includes both lifetime (“ever”) and past 30-day use measures for cigarettes and electronic vapor products, only past 30-day cigar use is assessed. Therefore, scoring could not account for lifetime cigar use, which might result in certain students receiving higher nicotine exposure scores than warranted. In addition, the 2025 national YRBS did not include measures of secondhand smoke exposure, which also could not be incorporated into scoring. An allocation of 100 points indicates more favorable practice and an allocation of 0 points indicates less favorable practice.

^¶^ The American Academy of Sleep Medicine recommends 8–10 hours of sleep per night for persons aged 13–18 years. Because the highest YRBS sleep response category is “10 or more hours,” students reporting >10 hours of sleep, which is above the recommended range, could not be distinguished from those reporting exactly 10 hours of sleep, which is within the recommended range. Under the American Heart Association’s scoring algorithm, sleep durations above the recommended range would receive lower scores; however, students in this response category were assigned the maximum score, which might have resulted in misclassification of the sleep health metric and overestimation of population sleep scores. An allocation of 100 points indicates more favorable practice and an allocation of 0 points indicates less favorable practice.

** BMI percentile calculation for YRBS data uses self-reported height, weight, sex, and age. CDC provides a SAS program that calculates BMI percentiles for children accounting for age and sex from CDC growth charts (https://www.cdc.gov/growth-chart-training/hcp/computer-programs/sas.html). Consistent with CDC guidance for datasets in which age is measured in years, students reporting their age as “12 years old or younger” or “18 years old or older” were assigned ages of 12 and 18 years, respectively. The formula for calculating ≥140% of the 95th percentile was added to the SAS program. Because evidence regarding the relationship between low BMI and CVH is limited and mixed, BMI <5th percentile was classified within the ideal BMI range (100 points). An allocation of 100 points indicates a more favorable score and an allocation of 0 points indicates a less favorable score.

An overall CVH score was calculated for each student as the mean of the five metric scores. Consistent with the AHA LE8 scoring approach, the five available metric scores were weighted equally. Individual metrics and the overall CVH score were categorized as high (80–100; favorable), moderate (50–79), or low (0–49) on the basis of LE8 cut points and accepted clinical thresholds. Demographic variables included sex (female or male), grade (9, 10, 11, or 12), and race and ethnicity (American Indian or Alaska Native, Asian, Black or African American [Black], Hispanic or Latino [Hispanic], Middle Eastern or North African [ME/NA], Native Hawaiian or Pacific Islander, White, or multiracial [selected more than one racial category]). Persons of Hispanic origin might be of any race but are categorized as Hispanic; all racial groups are non-Hispanic.

### Analysis

Among students who participated in the 2025 national YRBS, those with complete data for all five CVH metrics were included in the analytic sample. Students missing data for one or more CVH metrics were excluded. Item-level missing data resulted from student nonresponse, invalid responses, and, for some measures, differences in questionnaire administration. Weighted mean scores and 95% CIs were calculated for each CVH metric and for the overall CVH score. Weighted prevalence estimates and 95% CIs were calculated for high, moderate, and low categories of each metric and the overall CVH score. Estimates were calculated for the overall sample and stratified by sex, grade, and race and ethnicity.

All pairwise differences in mean scores and the prevalence of students in high score categories by sex, grade, and race and ethnicity were assessed using design-adjusted *t* tests. Statistical significance was defined as p<0.05. Corrections for multiple comparisons were not applied because the analyses were descriptive and hypothesis generating; each statistical test addressed a distinct research question and was interpreted independently. All estimates were calculated using Taylor series linearization. Mean and prevalence estimates with a denominator <30 were considered statistically unreliable and were therefore suppressed; thus, estimates among Native Hawaiian or Pacific Islander students are not presented. A sensitivity analysis was conducted to compare the distribution of sex, grade, and race and ethnicity between the analytic sample and excluded students to assess potential bias from missing data. All analyses were conducted using SAS-callable SUDAAN (version 11.0.4; RTI International) to account for the complex sampling design and weighting.

## Results

Among 10,710 students who participated in the 2025 national YRBS, a total of 5,192 (48%) had complete data for all five CVH metrics and were included in the analytic sample. A flow diagram describing exclusions that are due to missing data is provided (Supplementary Figure). Compared with excluded students, the analytic sample did not differ by sex or grade; however, a greater proportion of White students were included in the analytic sample (52.5%) than among those excluded (41.8%) (Supplementary Table 1).

The mean overall CVH score was 65.3, which falls within the moderate CVH score range (Table 2). Mean scores for individual CVH metrics ranged from 39.3 for diet (less favorable) to 85.4 for BMI (more favorable, indicating lower BMI). Scores were also high for nicotine exposure (80.5, indicating less exposure) and moderate for physical activity (62.0) and sleep health (59.1).

**TABLE 2:** Mean scores for cardiovascular health metrics and overall cardiovascular health* among high school students with complete data for all five metrics, by demographic characteristics — Youth Risk Behavior Survey, United States, 2025^†^

Characteristic	Overall CVH^§^	Diet^¶^	Physical activity**	Nicotine exposure^††^	Sleep health^§§^	Body mass index^¶¶^
	Mean (95% CI)	Mean (95% CI)	Mean (95% CI)	Mean (95% CI)	Mean (95% CI)	Mean (95% CI)
**Overall**	**65.3 (63.4–67.1)**	**39.3 (36.8–41.8)**	**62.0 (59.2–64.8)**	**80.5 (77.8–83.3)**	**59.1 (56.7–61.6)**	**85.4 (83.5–87.3)**
**Sex**						
Female	**64.2 (62.1–66.3)**	40.4 (37.1–43.7)	56.5 (53.3–59.7)	79.6 (76.3–82.9)	56.4 (54.0–58.9)	88.1 (86.1–90.1)
Male	**66.2 (64.4–68.0)**	38.3 (36.1–40.5)	66.9 (64.2–69.7)	81.4 (78.9–83.9)	61.5 (58.8–64.3)	83.0 (80.9–85.2)
**Grade**						
9	**68.3 (66.0–70.7)**	40.3 (37.7–42.9)	63.8 (60.5–67.2)	88.8 (85.3–92.2)	62.8 (59.1–66.5)	85.9 (83.2–88.5)
10	**65.9 (63.2–68.6)**	39.3 (36.2–42.5)	63.8 (59.9–67.8)	81.6 (77.5–85.7)	60.8 (57.0–64.6)	84.0 (80.9–87.1)
11	**63.7 (61.6–65.7)**	39.5 (36.4–42.6)	59.3 (55.2–63.4)	77.8 (73.1–82.6)	57.0 (54.0–60.1)	84.7 (81.8–87.6)
12	**62.7 (60.7–64.7)**	37.9 (34.5–41.2)	60.5 (57.6–63.4)	72.6 (68.6–76.5)	55.4 (52.2–58.6)	87.2 (85.3–89.0)
Race and ethnicity***^,†††^						
AI/AN	**62.3 (52.6–71.9)**	35.3 (22.3–48.3)	54.0 (37.6–70.3)	75.8 (53.7–97.9)	65.4 (42.6–88.2)	80.9 (61.4–100^§§§^)
Asian	**70.2 (68.0–72.4)**	48.7 (46.0–51.3)	60.0 (55.8–64.1)	92.5 (89.8–95.2)	56.0 (48.8–63.3)	93.7 (90.6–96.8)
Black or African American	**60.2 (58.9–61.5)**	33.7 (31.7–35.7)	53.4 (50.3–56.4)	81.4 (78.0–84.9)	51.8 (48.9–54.8)	80.8 (78.7–82.8)
Hispanic or Latino	**62.5 (60.7–64.4)**	37.6 (35.3–39.8)	55.8 (52.2–59.4)	79.8 (75.9–83.7)	58.2 (53.8–62.7)	81.2 (79.2–83.2)
ME/NA	**69.8 (61.6–78.0)**	53.8 (42.4–65.3)	62.4 (52.0–72.9)	87.1 (73.8–100^§§§^)	66.0 (53.5–78.6)	79.5 (69.5–89.6)
White	**66.8 (64.5–69.1)**	39.7 (36.2–43.2)	66.2 (63.0–69.4)	79.4 (75.9–82.9)	61.3 (58.6–64.0)	87.4 (85.0–89.8)
Multiracial	**65.8 (62.9–68.7)**	39.9 (34.6–45.2)	66.1 (61.5–70.6)	80.0 (73.6–86.4)	55.9 (51.9–60.0)	86.9 (82.6–91.1)

**Abbreviations:** AI/AN = American Indian or Alaska Native; CVH = cardiovascular health; NH/PI = Native Hawaiian or Pacific Islander; ME/NA = Middle Eastern or North African; YRBS = Youth Risk Behavior Survey.

* An overall CVH score was calculated for each student as the mean of the five metric scores.

^†^ N = 5,192 respondents with complete data for all five CVH metrics (complete case analysis). Students with missing data were excluded. The total number (N) of students answering each question varied. Data might be missing because 1) students in schools selected for both national and site-specific YRBS samples completed only one questionnaire; therefore, when the site-specific questionnaire was administered, it might not have included all questions on the national YRBS; 2) the student did not answer the question; or 3) the response was set to missing because of an out-of-range response or logical inconsistency. Mean estimates in each category are calculated based on nonmissing data.

^§^ As determined by *t *test analysis with Taylor series linearization (p<0.05), female students significantly differed from male students; grade 9 students significantly differed from grade 10, 11, and 12 students; grade 10 students significantly differed from grade 12 students; Asian students significantly differed from Black or African American (Black), Hispanic or Latino (Hispanic), White, and multiracial students; Black students significantly differed from Hispanic, ME/NA, White, and multiracial students; and Hispanic students significantly differed from White and multiracial students.

^¶^ As determined by *t* test analysis with Taylor series linearization (p<0.05), no significant differences were detected by sex or grade level; AI/AN significantly differed from Asian and ME/NA students; Asian students significantly differed from Black, Hispanic, White, and multiracial students; Black students significantly differed from Hispanic, ME/NA, White, and multiracial students; and Hispanic students significantly differed from ME/NA students.

** As determined by *t* test analysis with Taylor series linearization (p<0.05), female students significantly differed from male students; grade 9 students significantly differed from grade 11 and 12 students; grade 10 students significantly differed from grade 11 students; Asian students significantly differed from Black, ME/NA, and White students; Black students significantly differed from White and multiracial students; and Hispanic students significantly differed from White and multiracial students.

^††^ As determined by *t* test analysis with Taylor series linearization (p<0.05), no significant differences were detected by sex; grade 9 students significantly differed from grade 10, 11, and 12 students; grade 10 students significantly differed from grade 12 students; and Asian students significantly differed from Black, Hispanic, White, and multiracial students.

^§§^ As determined by *t* test analysis with Taylor series linearization (p<0.05), female students significantly differed from male students; grade 9 students significantly differed from grade 11 and 12 students; grade 10 students significantly differed from grade 12 students; Asian students significantly differed from Hispanic, ME/NA, and White students; and White students significantly differed from multiracial students.

^¶¶^ As determined by *t* test analysis with Taylor series linearization (p<0.05), no significant differences were detected by grade level; female students significantly differed from male students; Asian students significantly differed from Black, Hispanic, ME/NA, White, and multiracial students; Black students significantly differed from White and multiracial students; and Hispanic students significantly differed from White and multiracial students.

*** Persons of Hispanic origin might be of any race but are categorized as Hispanic; all racial groups are non-Hispanic.

^†††^ Mean estimates with a denominator <30 were considered statistically unreliable and were suppressed; therefore, estimates for NH/PI students are not presented.

^§§§^ CIs higher than the maximum of the theoretical score range of 0–100 were truncated at 100.

The mean overall CVH score was lower among female (64.2) than male students (66.2). Compared with male students, female students had lower mean scores for physical activity and sleep health but a higher (more favorable) BMI score. The mean overall CVH score was lower among students in grades 10 (65.9), 11 (63.7), and 12 (62.7) than among students in grade 9 (68.3), and scores were also lower among students in grade 12 compared with those in grade 10. Differences in scores across grades were observed for physical activity, nicotine exposure, and sleep health. Mean overall CVH scores varied by race and ethnicity: Asian (70.2), ME/NA (69.8), White (66.8), and multiracial (65.8) students had higher scores, whereas Black (60.2) and Hispanic (62.5) students had lower scores.

Overall, 20.7% of students had high, 61.3% had moderate, and 18.0% had low overall CVH scores (Figure 1) (Supplementary Table 2). The prevalence of students with high overall CVH scores was lower among female than male students (18.1% versus 23.0%) and lower among students in grades 10 (22.3%), 11 (16.4%), and 12 (15.3%) than among those in grade 9 (27.6%); students in grades 11 and 12 also had lower prevalences than students in grade 10. In pairwise comparisons by race and ethnicity, Black (11.2%) and Hispanic (14.9%) students had lower prevalences of high overall CVH scores than multiracial (21.0%), White (24.3%), and ME/NA (37.2%) students (Figure 2) (Supplementary Table 2).

**FIGURE 1:**
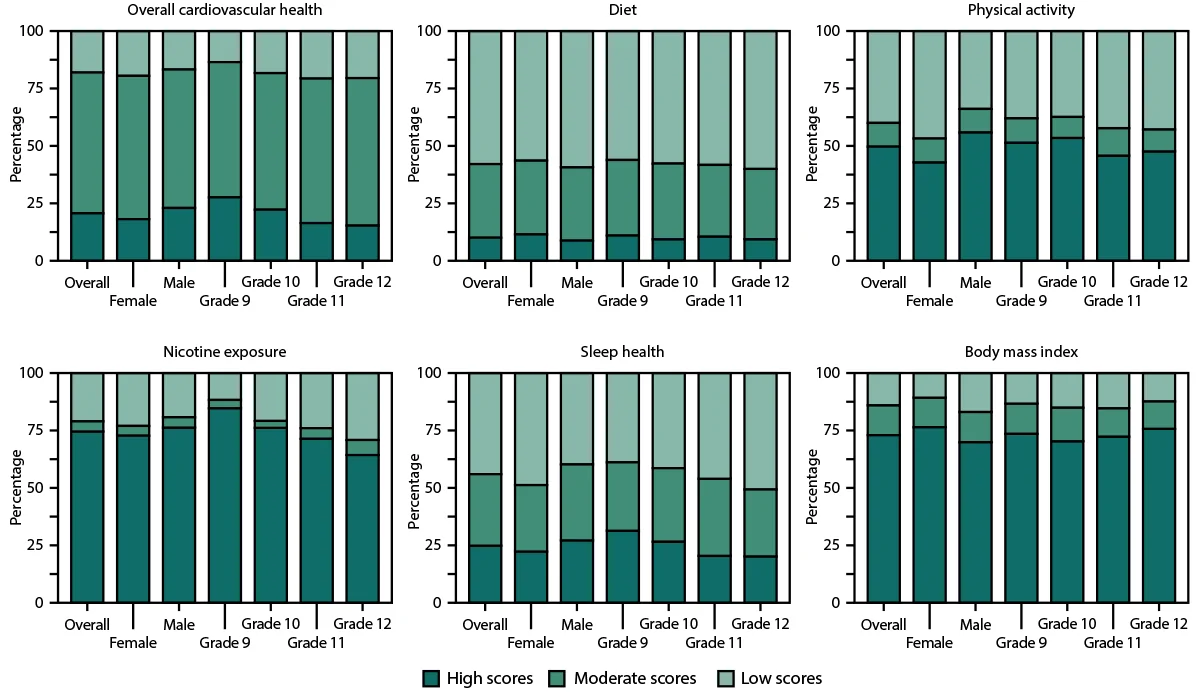
Prevalence of high, moderate, and low scores for cardiovascular health metrics and overall cardiovascular health,* overall and by sex and grade — Youth Risk Behavior Survey, United States, 2025 * The score categories and their associated values are low (0–49), moderate (50–79), and high (80–100). High scores are considered more favorable.

**FIGURE 2:**
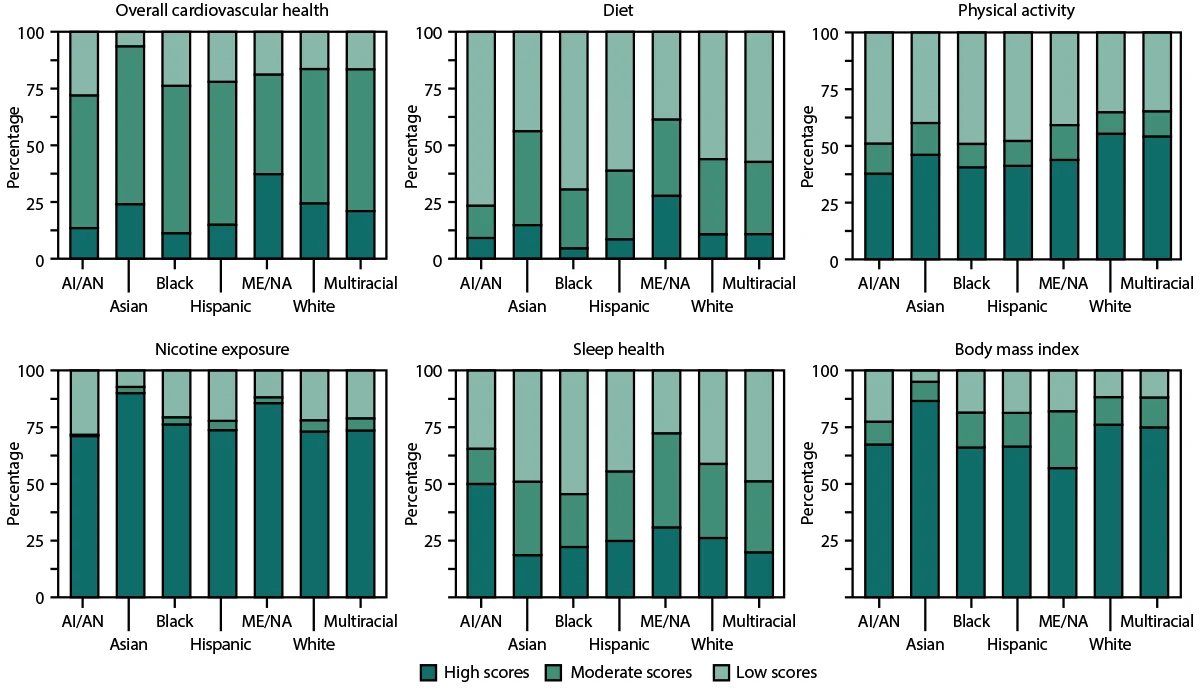
Prevalence of high, moderate, and low scores for cardiovascular health metrics and overall cardiovascular health,* by race and ethnicity — Youth Risk Behavior Survey, United States, 2025 **Abbreviations:** AI/AN = American Indian or Alaska Native; ME/NA = Middle Eastern or North African. * The score categories and their associated values are low (0–49), moderate (50–79), and high (80–100). High scores are considered more favorable.

## Discussion

This report demonstrates the usefulness of YRBSS for monitoring CVH among adolescents using a modified version of the AHA’s LE8 CVH score, adapted to the five metrics available in YRBSS. YRBSS provides a flexible and widely used surveillance platform for estimating these CVH metrics at national, state, territorial, tribal, and local school district levels. In this study using 2025 national data, approximately 80% of U.S. high school students had low or moderate overall CVH scores. Among the five CVH metrics, diet, sleep health, and physical activity had the least favorable scores, whereas BMI and nicotine exposure had the most favorable scores. These findings highlight potential areas for improvement across multiple modifiable health behaviors.

Certain YRBS-based CVH metrics approximated LE8 scoring because not all LE8 components were available in YRBS. However, the relative pattern of CVH metric scores and overall CVH scores in this 2025 YRBS study of high school students was similar to that reported for persons aged 2–19 years in AHA’s 2026 heart disease and stroke statistics update (*1*), which used 2013–2023 National Health and Nutrition Examination Survey (NHANES) data. NHANES provides a useful comparison because its measures align more closely with LE8. BMI (NHANES = 80.4; YRBS = 85.4) and nicotine exposure (NHANES = 86.5; YRBS = 80.5) scores were highest (more favorable), and diet score (NHANES = 43.9; YRBS = 39.3) was lowest (less favorable) in both surveys. The coexistence of relatively low diet scores with higher (more favorable) BMI scores might reflect both measurement and developmental factors. The BMI metric of LE8 assigns optimal scores to all adolescents below the 85th percentile (*4*), which might limit its ability to reflect less favorable dietary quality; in addition, dietary quality might influence cardiometabolic risk through pathways not immediately reflected in BMI, particularly during adolescence (*3*). The overall CVH score estimated using 2013–2023 NHANES data among persons aged 16–19 years was 73.2, compared with 65.3 estimated using 2025 YRBS data. Differences in absolute scores between YRBS- and NHANES-based estimates might reflect differences in measurement approaches, including the use of self-reported data in YRBS, differences in survey period, and the absence of clinical and biomarker-based metrics (e.g., blood pressure, blood lipid levels, and blood glucose levels) in this analysis. Nonetheless, the consistency in relative patterns across metrics supports the usefulness of YRBS data for monitoring key metrics of CVH.

The differences in overall CVH scores by sex, grade, and race and ethnicity observed in this report are also consistent with previous research (*7*,*10*,*11*). Female students had lower overall CVH scores than male students. Overall CVH scores were also lower among students in higher grades; students in grades 10, 11, and 12 had lower overall CVH scores than students in grade 9, suggesting that less favorable CVH is evident as early as grade 10. Lower physical activity and sleep health scores were also observed among female students and students in higher grades, along with lower nicotine exposure scores among students in higher grades. Black and Hispanic students had lower overall CVH scores than Asian, ME/NA, White, and multiracial students. The comparatively high prevalence of high overall CVH among ME/NA students warrants further examination. Limited research among U.S. adults of Middle Eastern origin has identified lower prevalence of selected CVD risk factors, including hypertension and obesity, relative to non-Hispanic White adults (*12*); however, findings across other risk factors and cardiovascular outcomes have been mixed. Evidence specific to ME/NA adolescents is sparse, and the wide CI for this estimate and heterogeneity within this population warrant cautious interpretation. More studies are needed to confirm this finding and examine factors that might contribute to CVH among ME/NA adolescents. Although the specific pairwise differences among racial and ethnic groups varied by metric, Black students had significantly lower scores for each individual CVH metric than students in at least one other racial or ethnic group. In addition, Hispanic students had significantly lower scores for each metric except sleep health than students in at least one other racial or ethnic group. These observed racial and ethnic differences might reflect the interaction of individual, developmental, interpersonal, community, and societal factors that influence health behaviors and access to resources differently across groups (*4*). These findings suggest that communities might experience different CVH-related challenges and needs and that prevention efforts should be designed to address the specific context in which they are implemented.

The findings in this report have implications for school and community policies and practices aimed at improving adolescent CVH. Because YRBSS is a school-based surveillance system, it provides an opportunity to examine CVH-related metrics within the settings where many adolescent health promotion policies and programs are implemented. Schools are uniquely positioned to influence health behaviors through coordinated policies, programs, and supportive environments. Strategies such as implementing and strengthening nutrition standards for school meals and competitive foods (i.e., foods and beverages sold outside school meal programs), offering taste tests of new menu items, providing free school meals for all students, and offering alternative breakfast models (i.e., “second chance” breakfast) can support healthy eating (*13*). Opportunities for regular physical activity, including access to physical education and participation in school- or community-based programs, can help students meet recommended activity levels (*7*,*14*). These opportunities can be part of a comprehensive school physical activity program that promotes physical activity before, during, and after school (*14*).

Through its Healthy Schools cooperative agreement (https://www.cdc.gov/healthy-schools/partners/index.html), CDC supports the implementation of evidence-based healthy eating and physical activity strategies across in-school and out-of-school settings. This initiative currently engages 20 state education and health agencies, universities, and tribes to strengthen policies and practices that promote student and staff member well-being within districts and schools. For example, Faribault Public School District in Minnesota introduced classroom physical activity carts^†^ in each school, expanded gymnasium access for high school students throughout the day, and facilitated community hiking initiatives. Similarly, Pattonville School District in Missouri (https://www.youtube.com/watch?v=MemgLJnI2d0) addressed healthy eating and food insecurity by establishing school gardens, greenhouse operations, and hydroponic systems and incorporating nutrition education that engages students in the “science of taste,” including approaches to reduce sodium intake such as substituting herbs and peppers in cooking. These initiatives demonstrate how educational settings use CDC support to cultivate healthier school environments.

Policies that support adequate sleep, including later school start times, along with education promoting consistent sleep schedules and reduced use of stimulating technology and screens at night, might improve sleep health among adolescents (*15*). In addition, comprehensive tobacco prevention and cessation efforts, including policies that address electronic vapor product use, can reduce nicotine exposure among adolescents (https://www.hhs.gov/sites/default/files/2024-sgr-tobacco-related-health-disparities-full-report.pdf). Incorporating CVH-related topics into school curricula aligned with the national health and physical education standards can further support student knowledge and skills related to healthy eating, physical activity, sleep, and tobacco prevention. Family and community engagement might help ensure that school-based strategies are relevant and can reinforce healthy choices and connect students with resources. Coordinated approaches that address multiple behaviors simultaneously might have the greatest impact on improving overall CVH.

## Limitations

General limitations for the YRBS are available in the overview report of this supplement (*9*). The findings in this report are subject to at least three additional limitations. First, the analytic sample was restricted to students with complete data for all five CVH metrics to ensure consistent calculation of the composite CVH score across all included participants. As a result, approximately 52% of students were excluded because of missing data. Missing data were concentrated in composite measures derived from multiple survey items (particularly diet and nicotine exposure) and reflected both student-level item nonresponse and site-level omission of survey items (*9*). Although the analytic and excluded samples did not differ by sex or grade, a greater proportion of White students were included in the analytic sample, which might affect the generalizability of findings. Second, the YRBS-based CVH metrics approximated LE8 metrics but were limited by the absence of certain components and health factors, including certain dietary components, secondhand smoke exposure, sleep durations >10 hours, blood pressure, blood lipid levels, and blood glucose levels. Although other studies among younger age groups have similarly lacked data for all LE8 metrics (*13*), these limitations might affect comparability with other studies and warrant caution when interpreting overall CVH scores. Finally, all measures were based on self-report and might be subject to reporting bias. Self-reported height and weight might result in underestimation of BMI (*16*). Although the anonymous, self-administered nature of the survey might improve reporting of sensitive behaviors among adolescents, certain misclassifications might remain.

## Future Directions

Future research can build on these findings by further examining factors associated with CVH among adolescents, including school, family, and community factors that might influence diet, physical activity, sleep, and nicotine use. Opportunities also exist to expand the use of YRBSS data to assess CVH at state, territorial, tribal, and local levels and to examine variation across jurisdictions. Linking CVH metrics with information on school and district policies and practices could help identify which strategies are associated with more favorable CVH metric scores and overall CVH scores among adolescents. Examining associations between CVH scores and school programs and policies related to nutrition, physical activity, sleep, and tobacco prevention could help public health and education agencies prioritize evidence-based interventions and allocate resources toward practices associated with improved adolescent CVH. Continued surveillance of CVH using YRBS can support the monitoring of trends over time and guide tailored, data-driven prevention and early intervention efforts in school-based health services and pediatric care settings.

## Conclusion

In 2025, approximately four in five U.S. high school students had a low or moderate CVH score. Among the five CVH metrics, diet, sleep health, and physical activity had the least favorable scores, highlighting potential areas for improvement during adolescence. These findings underscore the importance of promoting healthy behaviors early in life to support improved cardiovascular health in adulthood. Schools and communities can use these findings to strengthen policies and practices that support healthy eating, regular physical activity, adequate sleep, and avoidance of nicotine products. By using YRBS data to construct CVH metrics, this study establishes an approach for monitoring and studying adolescent CVH metrics over time and across national, state, territorial, tribal, and local school district populations.

## Footnotes

* 45 C.F.R. part 46.114; 21 C.F.R. part 56.114.

† Physical activity carts are mobile carts containing equipment and materials that teachers and staff members can use to facilitate classroom-based physical activity.
